# Use of *rbcL* and *trnL-F* as a Two-Locus DNA Barcode for Identification of NW-European Ferns: An Ecological Perspective

**DOI:** 10.1371/journal.pone.0016371

**Published:** 2011-01-26

**Authors:** G. Arjen de Groot, Heinjo J. During, Jan W. Maas, Harald Schneider, Johannes C. Vogel, Roy H. J. Erkens

**Affiliations:** 1 Ecology and Biodiversity Group, Institute of Environmental Biology, Utrecht University, Utrecht, The Netherlands; 2 Department of Botany, Natural History Museum, London, United Kingdom; French National Centre for Scientific Research, Université Paris-Sud, France

## Abstract

Although consensus has now been reached on a general two-locus DNA barcode for land plants, the selected combination of markers (*rbcL* + *matK*) is not applicable for ferns at the moment. Yet especially for ferns, DNA barcoding is potentially of great value since fern gametophytes—while playing an essential role in fern colonization and reproduction—generally lack the morphological complexity for morphology-based identification and have therefore been underappreciated in ecological studies. We evaluated the potential of a combination of *rbcL* with a noncoding plastid marker, *trnL-F*, to obtain DNA-identifications for fern species. A regional approach was adopted, by creating a reference database of trusted *rbcL* and *trnL-F* sequences for the wild-occurring homosporous ferns of NW-Europe. A combination of parsimony analyses and distance-based analyses was performed to evaluate the discriminatory power of the two-region barcode. DNA was successfully extracted from 86 tiny fern gametophytes and was used as a test case for the performance of DNA-based identification. Primer universality proved high for both markers. Based on the combined *rbcL* + *trnL-F* dataset, all genera as well as all species with non-equal chloroplast genomes formed their own well supported monophyletic clade, indicating a high discriminatory power. Interspecific distances were larger than intraspecific distances for all tested taxa. Identification tests on gametophytes showed a comparable result. All test samples could be identified to genus level, species identification was well possible unless they belonged to a pair of *Dryopteris* species with completely identical chloroplast genomes. Our results suggest a high potential of the combined use of *rbcL* and *trnL-F* as a two-locus cpDNA barcode for identification of fern species. A regional approach may be preferred for ecological tests. We here offer such a ready-to-use barcoding approach for ferns, which opens the way for answering a whole range of questions previously unaddressed in fern gametophyte ecology.

## Introduction

The development of universal DNA barcoding markers for land plants is challenging and the exact choice of loci has been heavily debated [1]–[3]. Recently, the Plant Working Group of the Consortium for Barcoding of Life decided on a standard two-locus barcode for all land plants, consisting of portions of the *rbcL* and *matK* plastid genes [4]. It was immediately emphasized that this core barcode might have to be augmented with supplementary loci in some groups due to lack of discriminatory power and/or primer universality. Ferns form one such group. While *rbcL* routinely has been used for studies on fern phylogeny (e.g., [5], [6]), species discrimination is sometimes insufficient [7], [8]. The generation of *matK* sequences for ferns is currently problematic, because this part of the chloroplast genome underwent a strong restructuring during the evolution of the fern clade [9]. None of the currently existing primer sets are likely suitable for all lineages of land plants [10], [11] and efforts are now focusing on the development of complex primer assays to achieve reliable amplification and sequencing of matK among land plants.

Nonetheless, while the feasibility and necessity of DNA barcoding for general taxonomic purposes has sometimes been criticized [12], [13], fern ecology is a typical example of a field for which its main goal, sample identification [14], would be of high practical and scientific value. Homosporous ferns have two free-living generations. The small and short-lived gametophytic generation plays an important role in the reproduction and dispersal ecology of ferns [15]. Many aspects of its ecology (e.g. tolerance to light and drought stress) differ radically from those of the sporophyte [16]. Studies on wild gametophyte populations and spore banks are therefore as essential for a proper ecological understanding of fern species as those on their sporophytes. Gametophytes however typically show very limited morphological complexity, making identification based on morphological features to species or even genus level often impossible [17]–[19]. Pteridologists are therefore in strong need of a proper alternative method for gametophyte identification.

Schneider & Schuettpelz [20] tested the principle of DNA barcoding of fern gametophytes using *rbcL* and successfully identified a cultivated gametophyte as *Osmunda regalis*. However, whether *rbcL* shows sufficient variation to allow general identification below genus level remains uncertain [8], [20]. Moreover, nowadays it is widely accepted that any valid plant barcode will be multi-locus, preferably existing of a conservative coding region like *rbcL*, in combination with a more rapidly evolving region, which is most likely non-coding [21]. The non-coding *trnL* intron and *trnL-F* intergenic spacer (IGS) have been repeatedly suggested for this purpose [4], [22], [23] and were successfully used by Li *et al.* [11] for identification of a mysterious aquatic gametophyte. Besides the technical issues of primer universality and sequence quality and complexity, Schneider & Schuettpelz [20] mentioned three potential difficulties for any tested marker to overcome: incomplete sampling of the online records to be used as a reference for identification (GenBank/EBI), the occurrence of misidentified and erroneous sequences in these online databases, and the potential inability of the marker to discriminate among species. An additional practical problem for fern gametophyte barcoding is the acquirement of sufficient DNA for multi-locus sequencing from minuscule samples.

We overcame the problems described above and created a reference database of trusted *rbcL* and *trnL-F* sequences, in this case for the ferns of NW-Europe. We then tested its potential in the context of primer universality and species discrimination power on a set of previously unidentified gametophyte samples originating from Dutch spore banks. This is the first test of the question whether the combination of *rbcL* and *trnL-F* (intron and IGS) possesses all the necessary qualities for standard DNA barcoding across a wide, complex variety of fern taxa. We herewith offer a ready-to-use approach for DNA barcoding of ferns, to be used in any fern ecological study, and show its strength when applied to a regional species pool.

## Results

### Database

A total of 77 *rbcL* sequences, belonging to 52 taxa, were selected and incorporated into the reference database (Table S1). For *trnL-F*, 74 sequences were selected, belonging to 47 taxa. New sequences were produced for a total of 26 species, for 13 species we had to rely completely on GenBank accessions. For a few species, only *rbcL* accessions were available from GenBank, which explains the difference in total number of included sequences between the two markers. Primer universality proved high for both *rbcL* and *trnL-F*. PCR amplification was successful for all tested taxa using the standard protocol, except for a herbarium sample of *Matteucia struthiopteris,* most likely due to poor DNA quality. PCR-protocols were robust. Sequencing success was >95% for *rbcL*, and equally high for *trnL-F* when using internal primers. The slightly higher effort needed for *trnL-F* in some taxa (mainly belonging to the families Blechnaceae and Thelypteridaceae) resulted from the presence of a homopolymer C-repeat in the intergenic spacer.

**Figure 1:**
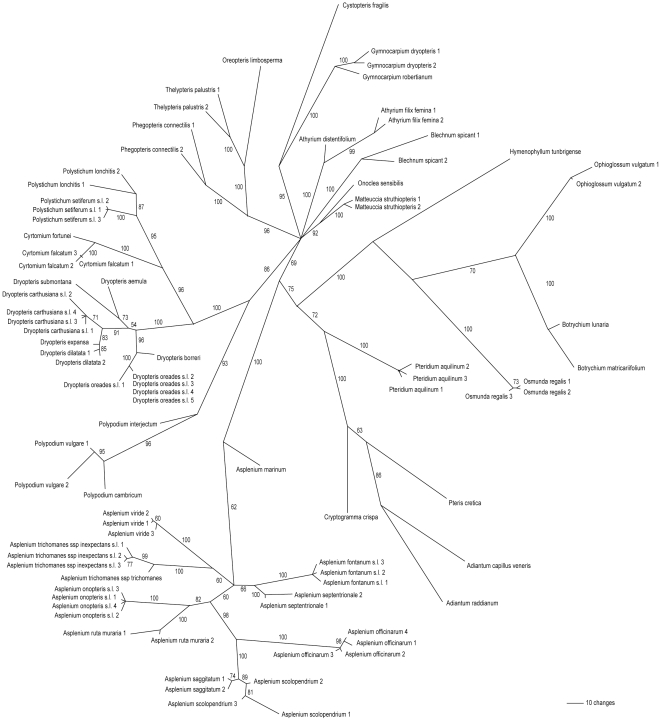
Bootstrap consensus tree of a maximum parsimony analysis of the combined *rbcL* and *trnL-F* dataset. Unrooted phylogram of the 50% majority rule bootstrap consensus tree from an analysis of the combined *rbcL* and *trnL-F* sequence data for 46 fern taxa occurring in NW-Europe. Bootstrap support values are given with each node. In case of multiple accessions per taxon, a sample number was added behind the taxon name.

Maximum parsimony analysis of the total dataset yielded the phylogram shown in Figure 1 (50% majority rule bootstrap consensus). Topology is mostly consistent with currently accepted pteridophyte phylogenies based on the same markers [6], [24], [25]. More importantly, 100% of the genera and 100% of the included species form their own well-supported monophyletic clade (bootstrap support >70%). Mean minimal interspecific P-distance per species (distance to nearest neighbour) was 0.031. Distances were however very skewed towards lower values, which is in line with general results for plastid loci in land plants [10]. Within genera, mean minimal interspecific P-distances were smallest for *Polystichum* (0.006) and *Dryopteris* (0.008). These genera had relatively short internal branches but still generally showed high bootstrap support (Figure 1).

**Figure 2:**
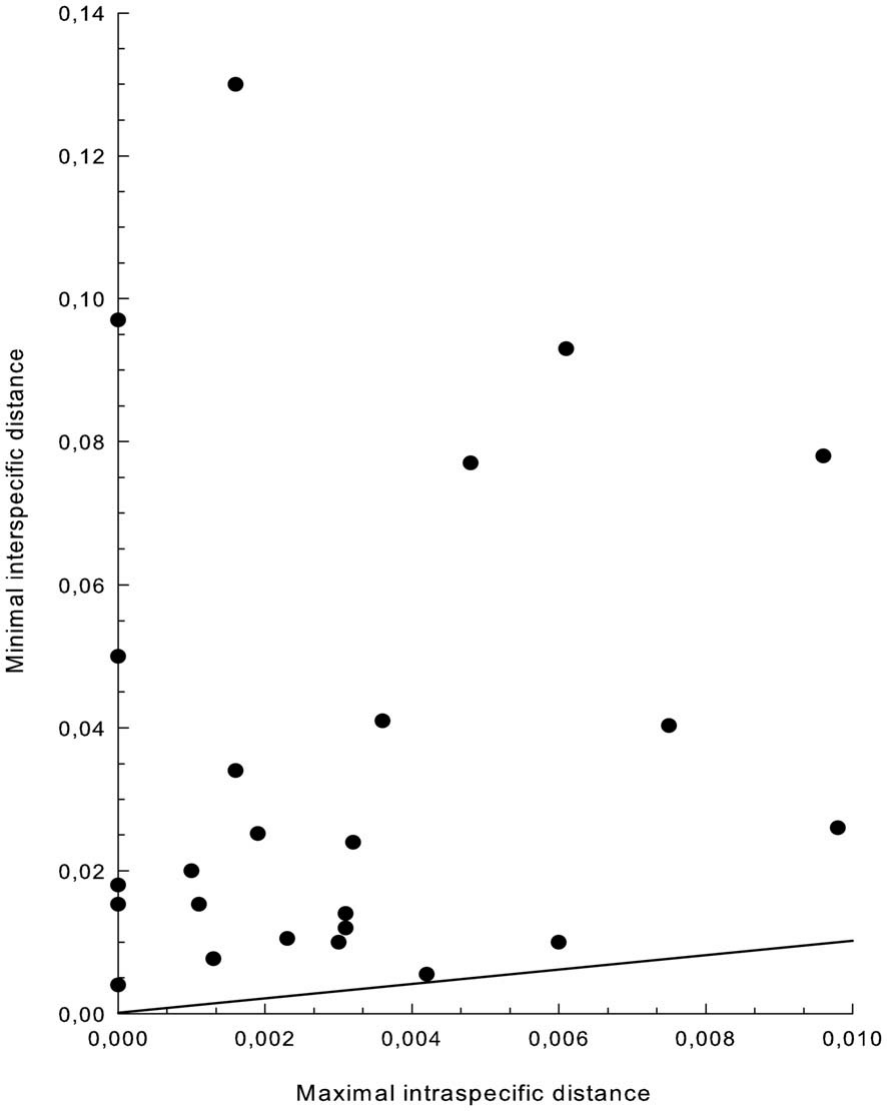
Sequence divergence among taxa. Sequence divergence across all 24 taxa for which sequences of multiple individuals were available. Divergence is given as the relation between the uncorrected maximal intraspecific and minimal interspecific P- distances. Along the black line both distance values equal each other.

Figure 2 presents an overview of sequence divergences based on a comparison between maximal intraspecific and minimal interspecific distances for all species for which multiple samples were included in the analysis. Exact values are given in Table S2. For all 24 taxa tested, minimal interspecific distances are clearly higher than maximal intraspecific distances. Not surprisingly, the smallest ratio was found for the subspecies of *A. trichomanes*, but even there minimal interspecific distance was 1.3 times bigger than maximal intraspecific distance.

### Case Study

To test the applicability of the generated database for fern species identification in ecological field studies, we used material of a total of 88 gametophytes which resulted from two different soil spore bank analyses [G.A. de Groot, unpublished data]. In one experiment soil samples were taken from different depths in four forests in the Dutch IJsselmeer polders. More than 25 fern species have been recorded for these forests, which were planted on the bare land of a former sea bottom and over the last decades acted as giant diaspore traps [26]. Samples were spread out in the greenhouse of the botanical gardens of Utrecht University and 86 of the resulting gametophytes were selected for analysis. Two additional gametophytes resulted from two different soil cores taken from the IJsselmeer lake bottom in spring 2008. Extraction of DNA from tiny amounts of gametophyte material proved well possible using a normal commercial extraction kit. Only two out of 88 attempts failed to produce a workable DNA solution. Table 1 gives an overview of the performance of sequencing and all necessary preceding steps. *rbcL* amplification was unproblematic and successful for all samples, in four random cases *trnL-F* amplification failed completely for unknown reasons. In some cases however, amplified bands were weak even after a nested PCR was performed and sequencing produced only a short fragment. Still, usable *rbcL* and *trnL-F* sequences were eventually produced for 97% and 99% of the samples respectively. Sequence coverage was around 84% for rbcL and 89% for *trnL-F*. The two tiny gametophyte samples extracted from lake-bottom sediments yielded usable DNA and were successfully sequenced.

**Table 1:** Overall performance of lab techniques.

Performance parameter:		Percentage (successful samples/total samples):	
Percent successful DNA isolation:		98	(86/88)
Percent amplification success:	*rbcL*	100	(86/86)
	*trnL-F*	93	(82/86)
Percent sequences obtained:	*rbcL*	97	(83/86)
	*trnL-F*	99	(80/82)
Percent sequence coverage:	*rbcL*	83	(N = 83)
	*trnL-F*	88	(N = 80)

Performance of used lab techniques, based on the analysis of 88 fern gametophyte samples from soil spore bank analyses. Percent sequences obtained: percentage of amplified fragments for which a sequence was obtained. Percent sequence coverage: mean percentage recovered data out of the total marker sequence length. N: number of included samples.

Identification using various methods classified the tested samples into six taxonomic groups. Identification results are given in Table 2. Identification at genus level succeeded for all samples using maximum parsimony analysis. 44 out of 79 samples with usable sequences of *rbcL* and *trnL-F* (56%) could easily be assigned to a single species. Maximum parsimony analyses using the *rbcL* + *trnL-F* reference database compiled in this study successfully identified samples as *Dryopteris dilatata*, *Athyrium filix-femina* or *Thelypteris palustris*. For *D. dilatata* and *A. filix-femina*, online BLASTn searches using *trnL-F* already successfully yielded the same identification, while searches using *rbcL* resulted in multiple options. BLAST analysis failed on samples identified as *T. palustris* in the phylogenetic analysis, either because of lack of resolution (*rbcL*) or because the species was not present in the online records (*trnL-F*). Two samples that were identified as *Polystichum setiferum* s.l. using the plastid markers could be identified as *P. aculeatum* using the additional *PgiC* marker. Parsimony analyses classified the remaining 35 samples (44%) as either *D. oreades* s.l. or *D. carthusiana* s.l. (see materials and methods for an explanation on used species groups); further identification to species name remained impossible using this method since the species have identical cpDNA. In a limited number of cases, bootstrap support turned out to be insufficient (bootstrap values 50–70%) to distinguish *D. dilatata* and *D. expansa* from *D. carthusiana* s.l. in the parsimony analyses (Table 2). *PgiC* was not able to resolve these complexes either: sequences of *D. carthusiana, D. dilatata* and *D. cristata* differ from *D. filix-mas* and *D. affinis*, but are identical among species in both combinations. The fact that BLAST results for *rbcL* or *trnL-F* do sometimes give only one or two options is simply due to lack of online accessions for the other species.

**Table 2:** Performance of identification methods.

		*Group I*	*Group II*	*Group III*	*Group IV*	*Group V*	*Group VI*
Final identification:		*D. filix-mas,*	*D. carthusiana*	*D. dilatata*	*A. filix-femina*	*T. palustris*	*P. aculeatum*
		*D. affinis,*	*or D. cristata*				
		*or D. oreades*					
Number of samples:		*25*	*10*	*9*	*23*	*10* *	*2*
BLAST *rbcL*	Identification:	*D. filix-mas,*	*D. carthusiana,*	*Dryopteris sp.*	*A. filix-femina*	*T. palustris*	*P. setiferum,*
		*D. affinis,*	*D. cristata,*		*A. distentifolium*		*P. lonchitis*
		*D. oreades*	*D. expansa*				
	MPI:	99.2	98.8	99.7	99.6	97.6	98.5
	Genus ID:	100.0	100.0	100.0	100.0	0.0	100.0
	Species ID:	0.0	0.0	0.0	4.3	100.0	0.0
BLAST *trnL-F*	Identification:	*D. filix-mas,*	*D. carthusiana*	*D. dilatata*	*A. filix-femina*	failure	*Polystichum sp.*
		*D. affinis*					
	MPI:	99.8	99.0	99.2	99.3	88.8	98.0
	Genus ID:	100.0	100.0	100.0	100.0	0.0	100.0
	Species ID:	0.0	0.0	100.0	100.0	0.0	0.0
Tree analysis (*rbcL* + *trnL-F*):	Identification:	*D. oreades* s.l.	*D.carthusiana* s.l.,	*D. dilatata*	*A. filix-femina*	*T. palustris*	*P. setiferum* s.l.
			*(D. dilatata),*				
			*(D. expansa)*				
	Bootstrap:	86.3	76.6	75.7	98.9	100.0	99.8
	Genus ID:	100.0	100.0	100.0	100.0	100.0	100.0
	Species ID:	0.0	0.0	100.0	100.0	100.0	0.0
*PgiC*	Identification:	*D. filix-mas,*	*D.carthusiana,*	*D.carthusiana,*	x	x	*P. aculeatum*
		*D. affinis*	*D.dilatata,*	*D.dilatata,*			
			*D.cristata*	*D.cristata*			
	Genus ID:	100.0	100.0	100.0			100.0
	Species ID:	0.0	0.0	0.0			100.0

Performance of various methods of molecular identification, tested on 79 samples for which usable *rbcL* and *trnL-F* sequences were obtained. Bootstrap: mean bootstrap value per sample, MPI: mean “maximum percent identity” in BLAST output, Genus/Species ID: Percentage of samples with valid identification to genus/species level. Failure: no valid identification using this marker.

*: including both samples from lake-bottom sediments.

## Discussion

### Selecting an Appropriate Barcode for Ferns

In line with previous results of for land plants in general [23] and for bryophytes [27], it proved possible to amplify and sequence *trnL-F* in ferns using a limited set of universal and very reliable primers, even when using suboptimal starting material. Primer universality of *rbcL* was already shown by various authors (e.g., [5], [6]) and is confirmed by our results. As in many plastid spacer regions in plants (e.g., [27]–[29]), a mononucleotide repeat of varying length is also present in fern *trnL-F* sequences. Such microsatellites potentially lead to reduced sequence quality and the use of any spacer region for plant DNA barcoding has therefore been criticized [30]. The use of a single pair of internal primers however successfully solved this problem in our case. Fazekas et al. [31], [32] showed that the use of proofreading enzymes fused to nonspecific dsDNA binding domains can also greatly improve sequence quality in case of mononucleotide repeats.

While Taberlet *et al.* [23] concluded that the *trnL* intron generally shows a low barcoding resolution among land plants, variation of the combined *trnL* and *trnL-F* spacer region appears to be surprisingly high for ferns. When combined with *rbcL*, both distance- and character-based tests showed a 100% resolving power at both genus and species level (Figure 1 and 2) for all included taxa with different chloroplast genomes. Although based on limited sample sizes, the calculated sequence divergences indicate the presence of a clear ‘barcoding gap’ across European fern taxa: enough difference between inter- and intraspecific distances to discriminate a species from its nearest neighbours [33]. Maximum parsimony analysis yielded the same well-supported differentiation, even though testing for it in a fundamentally different way. When adding this to its highly universal primers and robust PCR conditions, we conclude that *trnL-F* might be a valuable substitute for the problematic *matK* spacer [4], [10], and in combination with *rbcL* possesses al the necessary qualities to form a powerful barcode for species identification of pteridophytes, at least in NW-Europe.

The fern chloroplast genome is maternally inherited and hence non-recombining [34]. Recent allotetraploids therefore are likely to still share an identical chloroplast genome with the diploid parent that functioned as chloroplast donor or with a related polyploid sharing the same donor. This makes it often impossible to distinguish such related species based on the cpDNA, as sequences will be identical for any chloroplast marker. The same problem can be expected for apomictic aggregates. We explicitly chose to ignore this issue in our performance tests (see materials and methods), since plant DNA barcoding efforts are currently restricted to the single-copy cpDNA [4], [10], [35] and similar problems are thus to be expected for any candidate locus. The uniparental inheritance of the chloroplast is however an important issue to address, since this focus on the cpDNA can clearly reduce identification success in plant groups like temperate ferns, which are known for a high frequency of hybridization among closely related species [36] and a relatively frequent formation of apomictic lineages [37]. Application of a nuclear marker may often be useful for valid species discrimination in case of allopolyploid species, as well as for proper identification of hybrid individuals. We therefore suggest a further search for nuclear markers which are cheap and easy to apply. An example is the nuclear *PgiC* gene encoding cytosolic phosphoglucose isomerase [38]. *PgiC* has discriminatory power for European polystichoids, as shown by Bremer & De Groot [39]. They reported a simple identification method based on band lengths on an agarose gel, which discards the time and money consuming cloning step often discouraging barcoders from using nuclear loci [10]. However, for some other genera *PgiC* proved less useful, as discriminatory power was insufficient a. Clearly, more effort is needed to find alternative, more universal nuclear markers with sufficient discriminatory power.

### Methods for Species Assignment

We assessed both barcoding resolution of the tested markers and identity of the test samples by use of two different methods of comparison: a character-based parsimony analysis (checking for well-supported monophyly of species, with or without the presence of a query sequence) and a distance-based analysis (checking for (pair-wise) sequence similarity based on the number of identical nucleotide positions). Distance-based comparison methods are fast and can provide a rank list of nearest neighbours accompanied by a simple score of similarity [40]. This is useful when comparing samples with a limited reference database from which the correct species might be missing. Parsimony analysis might then yield a monophyletic clustering with the wrong species if other species are sufficiently different, while distance values will most likely still indicate a small percentage of dissimilarity. The other way around however, number of similar positions (similarity) is not always an indication of relatedness [41]: species with equal similarity scores might still be different at specific positions. Such character differences are taken into account when testing for well-supported monophyly based on parsimony. Additionally, the use of bootstrapping provides a measure of the expected mis-assignment due to local homoplasy [3]. Maximum parsimony analysis and bootstrapping are however computationally demanding, which might be a problem for barcoding applications. In our view however, both methods are complementary and we therefore advocate a joint use both in case of assessing the optimal set of barcodes and in case of actual species assignment tests by barcoding.

Proper sequence alignment is essential for any assignment method based on phylogenetic analysis, either distance- or parsimony-based [42]. Creating an acceptable global alignment above genus level is often impossible for loci with relatively high variation [2], [3], thus limiting the use of this type of methods for species assignment. An easy option is to keep problematic (highly diverging) sequences out of the alignment. In our case, sequences of five (evolutionary older) species could not be aligned with the rest, but the resolving power of the less-variable *rbcL* locus was already high enough to form well-supported monophyletic species. This once more indicates the strength of using a hierarchical multi-locus strategy with *rbcL* as a backbone locus, as proposed by the CBOL Plant Working Group [4]. In practice, a preliminary species assignment for most NW-European ferns will be already possible based on *rbcL* and might be verified or specified by comparison with *trnL-F* sequences. This can either be done manually, by use of pair-wise distance analysis, or by performing a second parsimony analysis using data of a single genus only. At the same time, current advances in comparative genomics induce innovations in bioinformatic techniques for automated analysis of large and diverse sequence databases [43]. Newly developed tools for (multiple) sequence alignment are more dynamic, using adjustable local algorithms and instant global replotting, and as such are better suited for alignment of distantly related sequences [44], [45].

### Application of DNA Barcoding in Fern Ecology

In part of the BLAST searches performed for test sample identification, MPI values were equally high for some Asian and American species as for the (more likely) European species. Such species were specifically ignored here, but these results indicate that DNA barcoding of ferns might be more problematic on a global scale. Testing the rbcL + trnL-F barcode on a wider scale and/or a further search for (additional) fern barcodes cannot be avoided. Finding universal primers for the exhaustive variation in closely related fern species will be extremely difficult. For ecological applications a regional barcoding approach might therefore be the best choice, as its goal is simple: finding the most likely identification for a specimen encountered, given its local environment. For this purpose, a regional approach is most efficient, as it enables the use of a restricted reference database of trusted sequences for all species of a specific region or ecological community [21], [46]. Our study approached such a database for the native ferns of North-western Europe. We acknowledge that restricting the reference set to a certain region neglects the possibility of invasions and garden escapes (although typical examples might be included in the database), but the same is true for common practice in morphological identification.

Even though 44% of the tested samples could not be resolved to a single species because they belonged to one of the polyploidy complexes described above, all samples could be resolved to genus level. The fact that even difficult samples with low quantities of degraded DNA, like gametophytes resulting from spores of the long-term lake-bottom spore bank, could successfully be sequenced and identified opens the way for answering a whole range of previously ignored questions in the field of fern gametophyte ecology. Gametophytes derived from samples of the deeper soil layers typically were small and unhealthy. Such individuals would never have produced sporophytes of identifiable size, thus making them unidentifiable by conventional morphological methods. However, these gametophytes represent the few long-term surviving spores in the soil and potentially yield valuable information about past vegetation composition and diversity. Likewise, morphological identification is inapplicable when studying population biology or reproductive success of gametophytes in the field. In such cases barcoding is a very efficient and valuable technique. Already, some ecologists used a barcoding approach to identify a specific unknown plant sample for practical purposes [11], [47], [48]. We now offer a complete and ready-to-use approach for wider application of DNA barcoding in ecological studies on ferns in north-western Europe.

## Materials and Methods

### Sequence Database for DNA Barcoding

#### Taxon sampling and origin of sequences

52 taxa, representing 23 genera, were included in our reference database and together cover the diversity of terrestrial ferns occurring and sporulating in the wild in North-western Europe. This region harbours the taxa most likely to reach the area from which the samples of our case study (see below) originated, and as such is a typical example of a regional approach to be used by ecologists. We defined “North-western Europe” as comprising the British Isles, The Netherlands, Belgium, Luxemburg, Northern France and Western Germany (thus excluding the Nordic countries), and a species list for this area was compiled from Stace [49], Lambinon *et al.* [50] and Van der Meijden [51]. Fresh water ferns and horsetails were excluded since they fell outside the scope of the spore bank studies for which the database was originally developed. In order to obtain a set of trusted reference sequences, we used self-produced sequences for each taxon whenever possible. Sequences were either obtained from previous studies of the authors or sequenced *de novo* from freshly collected leaf material or herbarium specimens. Identifications were checked by experienced fern taxonomists. GenBank accessions were used for a small number of taxa for which no properly identified material was available. Additional GenBank accessions were added to the database when available in order to represent multiple individuals per species. A full list of sequences present in the database and their origin is given in Table S1.

#### DNA extraction, amplification and sequencing

Freshly collected specimens were stored on silica prior to extraction. DNA was extracted using the GenElute™ Plant Genomic DNA Miniprep Kit (Sigma-Aldrich, St. Louis, USA) following the manufacturer's protocol. We amplified two plastid regions, a c.1300 bp. long fragment of the *rbcL* gene (using 1FN and 1361R; Table 3) and a c. 900 bp. long combination of the *trnL* gene and *trnL-trnF* intergenic spacer together referred to as the *trnL-F* region (using FERN-1 and ‘f’; Table 3). For samples with problematic amplification we additionally used internal primers (Table 3). For *rbcL*, this concerned some seemingly random samples, for *trnL-F* a few taxa were inherently difficult because a homopolymer C-repeat was present in the intergenic spacer.

**Table 3:** Standard primers for amplification and sequencing.

Marker:	Primer:	Use:	Sequence (5′ to 3′):	Reference:
*rbcL*	1FN	F, AS	ATGTCACCACAAACRGAGACTAAAGC	This study
	424F	F, AS	CTGCTTATTCTAAGACTTTC	[52]
	878F	F, AS	TCATCGTGCAATGCATGC	[52]
	432R	R, A	ATAAGCAGGAGGGATTCGCAGATC	[52]
	940R	R, A	CATGCGTAATGCTTTGGCCAA	[53]
	1361R	R, AS	TCAGGACTCCACTTACTAGCTTCACG	[54]
*trnL-F*	FERN-1	F, AS	GGCAGCCCCCARATTCAGGGRAACC	[55]
	720F	F, AS	CCGTGAGGGTTCRANTCCCCTCTAT	This study
	743R	R, A	ATAGAGGGANTYGAACCCTCACGG	This study
	f	R, AS	ATTTGAACTGGTGACACGAG	[22]
PgiC	14F	F, AS	GTGCTTCTGGGTCTTTTGAGTG	[36]
	16R	R, A	GTTGTCCATTAGTTCCAGGTTCCCC	[36]
	14FN	F, AS	TGGGTCTTTTGAGTGTTTGG	This study
	16RN	R, A	CATTGCTTTCCATACTA	This study

F: forward primer; R: reverse primer. A: used for amplification; AS: used for amplification and sequencing. For PgiC, primers 14F and 16R were used on *Dryopteris*, primers 14FN and 16RN were used on *Polystichum* species.

DNA amplification was performed in 25 µl reactions containing 1× buffer, 3.5 µM MgCl_2_, 0.1 µM primers, 200 µM dNTP, 1% BSA and DMSO, 0.25 U RedTaq™ polymerase (Sigma-Aldrich, St. Louis, USA) and 1.5 µl of DNA template. Thermal cycling conditions for *rbcL* were: 50 s at 96°C, 30 cycles of 50 s at 96°C, 50 s at 53°C and 90 s at 72°C, and a final extension of 7 min at 72°C. For *trnL-F* the following protocol was used: 5 min at 95°C. 30 cycles of 5 s at 95°C, 30 s at 53°C and 90 s at 72°C, and a final step of 10 min at 72°C.

PCR products were purified on a 96-wells plate (Thermo Fisher Scientific, Waltham, USA) using gel filtration with Sephadex™ G-50 (GE Healthcare, Uppsala, Sweden). Sequencing was performed by Macrogen (Seoul, Korea and Amsterdam, The Netherlands) using the amplification primers (except for samples amplified with internal primers, which were short enough to sequence with forward primers only). All obtained sequences are available in GenBank (accession numbers listed in Table S1).

#### Sequence alignment and data analysis

Sequences were edited and assembled in SeqMan 4.0 (DNAStar Inc., Madison, USA) and manually aligned using PAUP* 4.0b10 [56]. Large indels were coded using simple indel coding as described by Simmons & Ochoterena [57].

To estimate species discrimination based on the chosen markers, we used a combination of two complementary measures: a comparison of interspecific and intraspecific distance values and a tree-based analysis. The tree-based strategy was performed using maximum parsimony rather than using a neighbour joining approach, since besides pure distance values also specific shared characters can prove useful in discriminating different lineages for barcoding purposes [44], [58]. Most-parsimonious trees were generated in PAUP* 4.0 [56] using random taxon additions, TBR swapping and equal weights. Heuristic bootstrap analysis [59] was performed with 1000 bootstrap replicates, 100 random addition cycles per bootstrap replicate, TBR swapping and equal weights. Species discrimination success was then based on monophyly: a species was successfully resolved when forming a monophyletic group with sufficient bootstrap support (bootstrap value >70%). Uncorrected minimal interspecific P-distances were calculated for all included species. Species discrimination based on distance values was tested for all species with multiple sequences in the database, according to CBOL guidelines [34]. Discrimination was considered successful if the minimal interspecific distance involving a species was larger than its intraspecific distance [4].

As previous studies showed that the resolving power of multi-locus barcodes is almost always higher than that of single-locus barcodes [3], [4], [60], [61], all testing was based on the combined dataset of *rbcL* and *trnL-F*. Four taxa (*Botrychium lunaria*, *Botrychium matricariifolium*, *Ophioglossum vulgatum* and *Osmunda regalis)* could not be properly included in the *trnL-F* alignment due to large sequence divergence (differences at so many positions that any alignment would be unreliable). Evidence for monophyly of these species thus was based on *rbcL* sequences only, but P-distances could be calculated from the total dataset.

As explained in the discussion, recent polyploids may share their complete chloroplast genome with their diploid chloroplast donor or with a related polyploid originating from the same donor, which makes them undistinguishable by any cpDNA marker. The same problem is seen in apomicts. Our database includes several examples of such complexes. *Dryopteris cristata* (L.) A. Gray and *D. carthusiana* (Vill.) H.P. Fuchs belong to a complex of allotetraploids sharing ancestral diploid genomes [62]. Stein et al. [63] showed that both species have identical cpDNA, which they most likely inherited from a shared extinct parent called *D. semicristata* [64]. The tetraploid *Dryopteris filix-mas* (L.) Schott is closely entangled with the apomictic *D. affinis* (Lowe) Fraser-Jenk [65] aggregate and the species presumably share parents, one of which being *D. oreades* Fomin [66], [67]. *Polystichum aculeatum* (L.) Roth is an allotetraploid derivative from the cross between *P. setiferum* (Forssk.) Woynar, which donated the cpDNA, and *P. lonchitis* (L.) Roth [68]. *Asplenium adiantum-nigrum* L. is interpreted as a segmental allotetraploid (at least in Europe) that involved *A. onopteris* L as the maternal parent [69]. Finally, *A. fontanum* is most likely donor of the cpDNA to *A. foreziense* [70]. For a proper analysis of the discriminatory power of the chosen markers, we chose to collapse the taxonomy in each of these cases and name each member of the group after the (also included) diploid cpDNA donor [61]. For *D. cristata* and *D. carthusiana* the donor could not be included and their sequences were instead named *D. carthusiana* s.l.. An overview of the performed merges can be found in Table S3.

### A Case Study: Identification of Fern Gametophytes from Spore Bank Analyses

#### Sample processing

We used material of a total of 88 gametophytes which resulted from two different soil spore bank analyses [G.A. de Groot, unpublished data]. Gametophytes ranged in size from c. 1 mm^2^ to 1.5 cm^2^. The smallest gametophytes resulted from the deeper forest soil layers (>15 cm) and lake bottom samples, and were sampled for DNA extraction at small size because they didn't grow any further and started to look unhealthy. Selection was based on an optimal sampling scheme for the spore bank analysis, but we made sure that the observed morphological variation (mainly presence of glandular hairs) was covered. Selected gametophytes were rinsed with water to avoid contamination and dried on silica in a 1.5 ml Eppendorf tube. Because of the very small size of the gametophytes, it was impossible to use only part of the material and use the rest for further culturing. Instead, complete individuals were grinded with mortar and pestle and a tiny amount of sand, after which DNA was extracted using the GenElute™ Plant Genomic DNA Miniprep Kit (Sigma-Aldrich, St. Louis, USA) following the manufacturer's protocol, but using 10% of all volumes and a final elution in 30 µl water [71]. For 86 out of 88 gametophytes this resulted in dissolved DNA of adequate concentration to be used in multiple PCR reactions. We prefer this method above tissue-direct PCR [72] since in case of tiny samples the latter leaves no option for retrial or application of multiple markers. *rbcL* and *trnL-F* were amplified in respectively three and two parts using the primers listed in Table 3 (with the earlier described reaction mixture and cycling protocol) and sequenced by Macrogen (Seoul, Korea) using the forward primers. Acquired sequences were edited and assembled in SeqMan 4.0 (DNAStar Inc., Madison, USA).

#### Species identification

BLASTn searches were applied to all produced sequences using the available online databases (i.e. GenBank, EMBL). Since a considerable part of the existing online accessions involved partial sequences, BLAST results were ranked by maximal percent identity (MPI) instead of maximal bit core. Since here we specifically test a regional approach for ecological purposes and non-European species were considered unlikely to be the correct identification, such species were ignored in all output. Identification at genus level was considered successful when all hits with MPI scores >95% involved a single genus. Species identification was considered successful only when the highest MPI included a single European species and scored above 95%.

Additionally, all sequences were manually aligned with the above described reference database using PAUP* 4.0b10 [55]. Heuristic parsimony analyses using the combined *rbcL* and *trnL-F* database were performed separately for every unknown gametophyte by including one individual at a time in the analysis. Bootstrapping [58] was performed with 20 bootstrap replicates, 100 random addition cycles per bootstrap replicate, TBR swapping and equal weights. Genus identification was considered successful when the unknown gametophyte formed a monophyletic group together with all members of a single genus, with a bootstrap support >70%. An equal strategy was applied for identification at species level.

Two samples could only be identified as *Polystichum setiferum* s.l. using above described methods since *P. setiferum* and *P. aculeatum* (both occurring in the polder forests) share the same cpDNA. The nuclear *PgiC* gene for cytosolic phosphoglucose isomerase [37] was applied to discriminate up to species level, following a method based on band size differences described by Bremer & De Groot [38]. The length of the amplified fragment for *PgiC* differs between *Polystichum lonchitis* and *Polystichum setiferum*, resulting in a difference in band lengths on agarose gel between the two species, and a double banding pattern in their hybrid derivative, *Polystichum aculeatum*. These patterns easily discriminate the three species and can be used for identification of unknown samples. New primers were developed for this purpose (Table 3). Thermal cycling protocols were copied from Ishikawa *et al.* [37].

## Supporting Information

Table S1 ***rbcL*** **and** ***trnL-F*** **sequence information.** Sequence origin (O = sequenced by authors in previous study, G = downloaded from Genbank, N = sequenced for this study), voucher information (herbarium, collection number. EB = Ecology & Biodiversity Group, Utrecht University voucher collection), collector, publication information and Genbank accession numbers of the *rbcL* and *trnL-F* sequences utilized for this study.
(DOC)

Table S2 **Maximal intraspecific genetic distances, minimal genetic distances towards the nearest neighbour and their ratio.** Data are presented for all taxa with multiple individuals in the dataset. Ratios are calculated as maximal interspec. distance/minimal intraspec. distance. All distances are uncorrected P-distances based on the combined *rbcL* and *trnL-F* sequence data. N = number of individuals in the dataset.
(DOC)

Table S3 **Used species complexes.** Overview of species merged into a single complex because of identical chloroplast genomes. All sequences were named after the diploid cpDNA donor, unless this donor could not be included (*Dryopteris “semicristata”*, see text).
(DOC)
