# Supplementary material for: Use of rbcL and trnL-F as a Two-Locus DNA Barcode for Identification of NW-European Ferns: An Ecological Perspective
Source: PLoS One. 2011 Jan 26;6(1):e16371. doi: 10.1371/journal.pone.0016371 (PMC3027654; doi:10.1371/journal.pone.0016371)
Supplement: Table S1 — rbcL and trnL-F sequence information. Sequence origin (O = sequenced by authors in previous study, G = downloaded from Genbank, N = sequenced for this study), voucher information (herbarium, collection number. EB = Ecology & Biodiversity Group, Utrecht University voucher collection), collector, publication information and Genbank accession numbers of the rbcL and trnL-F sequences utilized for this study. (DOC) [file pone.0016371.s001.doc]

| **rbcL** | | | | | |
| --- | --- | --- | --- | --- | --- |
| **Taxon:** | **Origin:** | **Voucher:** | **Collector:** | **Publication:** | **Accession:** |
| *Adiantum capillus-veneris* | G | PE, G.Zhang s.n. | G. Zhang | 1 | DQ432659 |
| *Adiantum raddianum* | G | UTC, #244 | Wolf | 2 | U05906 |
| *Asplenium fontanum 1* | O, N | EB, #863 | Acock | This paper | HQ676492 |
| *Asplenium fontanum 2* | O | BM, F-3-92 | Vogel | 3 | AF525268 |
| *Asplenium foreziense* | O, N | EB, #920 | Bond | This paper | HQ676493 |
| *Asplenium marinum* | O | BM, MAR-5 | Vogel | 3 | AF240647 |
| *Asplenium officinarum 1* | O | EB, # 707 | Van De Riet | This paper | HQ676494 |
| *Asplenium officinarum 2* | O | BM | Vogel | - | AF240643 |
| *Asplenium onopteris 1* | O | SAR, Schneider s.n. | Schneider | 4 | AY300131 |
| *Asplenium onopteris 2* | G | RV8094 | Viane | 5 | GU586792 |
| *Asplenium ruta-muraria 1* | O, N | EB, #712 | De Groot | This paper | HQ676495 |
| *Asplenium ruta-muraria 2* | O | BM, RUT-16 | Vogel | 3 | AF525273 |
| *Asplenium sagittatum 2* | O | BM, SAG-1 | Vogel | 3 | AF240646 |
| *Asplenium scolopendrium 1* | O | EB, #309 | De Groot | This paper | HQ676496 |
| *Asplenium scolopendrium 3* | O | BM, SCOL-73 | Vogel | 3 | AF240645 |
| *Asplenium septentrionale 1* | O | BM, SEPT-17 | Vogel | 3 | AF525275 |
| *Asplenium septentrionale 2* | G | HEID | Schulze | 6 | AF318586 |
| *Asplenium trichomanes ssp. quadrivalens 1* | G | HEID | Schulze | 6 | AF318595 |
| *Asplenium trichomanes ssp. quadrivalens 2* | O | BM, Q-272 | Vogel | 7 | AY549744 |
| *Asplenium trichomanes ssp. trichomanes* | G | HEID | Schulze | 6 | AF318594 |
| *Asplenium trichomanes ssp. inexpectans* | O | BM, I-46-B04 | Vogel | 7 | AY549743 |
| *Asplenium viride 1* | O | BM, JCV 1334 | Vogel | 7 | AY549734 |
| *Asplenium viride 3* | G | HEID | Schulze | 6 | AF318593 |
| *Athyrium distentifolium* | G | GOET, Schuettpelz 536 | Schuettpelz | 8 | EF463304 |
| *Athyrium filix femina 1* | O, N | EB, #306 | De Groot | This paper | HQ676497 |
| *Athyrium filix femina 2* | G | - | - | 9 | EU329032 |
| *Blechnum spicant 1* | O, N | EB, #911 | De Groot | This paper | HQ676498 |
| *Blechnum spicant 2* | G | - | - | - | AB040571 |
| *Botrychium lunaria* | G | - | - | - | DQ849146 |
| *Botrychium matricariifolium* | G | - | - | 10 | L40967 |
| *Cryptogramma crispa* | O | TUR, Christenhusz 3871 | Christenhusz | 11 | EF452148 |
| *Cyrtomium falcatum 1* | O, N | EB, #972 | De Groot | This paper | HQ676499 |
| *Cyrtomium falcatum 2* | G | VT, D.P.Little 342 | Little | 12 | AF537226 |
| *Cyrtomium falcatum 3* | G | KUN, LJM 059 | Lu | 13 | AY694796 |
| *Cyrtomium fortunei* | G | IND | Mitsuta | 12 | AF537227 |
| *Cystopteris fragilis* | O, N | EB, #912 | De Groot | This paper | HQ676500 |
| *Dryopteris aemula* | G | - | - | 14 | AY268881 |
| *Dryopteris affinis 1* | O, N | EB, #706 | De Groot | This paper | HQ676501 |
| *Dryopteris affinis 2* | G | COLO, 11824 | Crabbe | 14 | AY268849 |
| *Dryopteris borreri* | G | COLO | Vasak | 14 | AY268847 |
| *Dryopteris carthusiana 2* | G | - | - | 14 | AY268883 |
| *Dryopteris carthusiana 3* | G | COLO, 9327 | Argus | 14 | AY268846 |
| *Dryopteris dilatata 2* | G | COLO, 679 | Krasnobovov | 14 | AY268848 |
| *Dryopteris expansa* | G | COLO, 7921 | Nelson | 14 | AY268844 |
| *Dryopteris filix mas 1* | O, N | EB, #305 | De Groot | This paper | HQ676502 |
| *Dryopteris filix mas 2* | G | COLO, 1421 | Hogan | 14 | AY268845 |
| *Dryopteris oreades* | G | COLO | Vasak | 14 | AY268850 |
| *Dryopteris submontana* | O | BM | Vogel | - | AF240653 |
| *Gymnocarpium dryopteris 1* | O, N | EB, #304 | De Groot | This paper | HQ676503 |
| *Gymnocarpium dryopteris 2* | G | UTC, Wolf 238 | Wolf | 2 | U05925 |
| *Gymnocarpium robertianum* | O, N | EB, #472 | De Groot | This paper | HQ676504 |
| *Hymenophyllum tunbrigense* | G | P, Hennequin 2004-2 | Hennequin | 15 | EU553547 |
| *Matteuccia struthiopteris 1* | G | TI, CT1004 | Tsutsumi | 16 | AB232415 |
| *Matteuccia struthiopteris 2* | G | IND, Jensen s.n. | Jensen | 17 | U62032 |
| *Onoclea sensibilis* | G | UTC, MBG791216 | - | 2 | U05936 |
| *Ophioglossum vulgatum 1* | O, N | EB, #471 | De Groot | This paper | HQ676505 |
| *Ophioglossum vulgatum 2* | G | Muola s.n. | Muola | 18 | DQ026595 |
| *Oreopteris limbosperma* | O, N | TUR, Christenhusz 3719 | Christenhusz | This paper | HQ676506 |
| *Osmunda regalis 1* | G | - | - | - | AB076259 |
| *Osmunda regalis 2* | G | - | - | - | AB024948 |
| *Osmunda regalis 3* | G | - | - | - | AB076258 |
| *Phegopteris connectilis 1* | O, N | EB, #308 | De Groot | This paper | HQ676507 |
| *Phegopteris connectilis 2* | G | UC, Cranfill s.n. | Cranfill | 19 | AF425179 |
| *Polypodium cambricum* | O | BM |  | 20 | FJ825703 |
| *Polypodium interjectum* | O, N | BM, Christenhusz 390 | Christenhusz | This paper | HQ676508 |
| *Polypodium vulgare 2* | O | GOET, Schneider s.n. | Schneider | 21 | EF551065 |
| *Polystichum aculeatum* | O, N | EB, #303 | De Groot | This paper | HQ676509 |
| *Polystichum setiferum 1* | O, N | EB, #700 | De Groot | This paper | HQ676510 |
| *Polystichum setiferum 2* | G | VT, ex. hort. | - | 12 | AF537254 |
| *Polystichum lonchitis 1* | O, N | EB, #919 | De Groot | This paper | HQ676511 |
| *Polystichum lonchitis 2* | G | VT, D.P.Little 344 | Little | 12 | AF537247 |
| *Pteridium aquilinum 1* | O, N | EB, #908 | Larssen | This paper | HQ676512 |
| *Pteridium aquilinum 2* | O | BM, PTER-1 | Vogel | 4 | AY300097 |
| *Pteridium aquilinum 3* | G | UTC, Wolf 237 | Wolf | 2 | U05939 |
| *Pteris cretica* | G | DUKE | - | 11 | EF452170 |
| *Thelypteris palustris 1* | O, N | EB, #909 | Villaret | This paper | HQ676513 |
| *Thelypteris palustris 2* | G | UTC, MBG810566 | - | 2 | U05947 |
|  |  |  |  |  |  |
| **trnL-F** | | | | | |
| **Taxon:** | **Origin:** | **Voucher:** | **Collector:** | **Publication:** | **Accession:** |
| *Adiantum capillus-veneris* | G | PE, G.Zhang s.n. | G. Zhang | 1 | DQ432689 |
| *Asplenium adiantum-nigrum 1* | O, N | BM, ADI-35 | - | This paper | HQ676539 |
| *Asplenium adiantum-nigrum 2* | O, N | BM, ADI-46 | Spencer | This paper | HQ676540 |
| *Asplenium fontanum 1* | O, N | EB, #863 | Acock | This paper | HQ676514 |
| *Asplenium fontanum 2* | O | BM, F-3-92 | Vogel | 3 | AF525239 |
| *Asplenium foreziense* | O, N | EB, #920 | Bond | This paper | HQ676515 |
| *Asplenium marinum* | O | BM, MAR-5 | Vogel | 3 | AF240662 |
| *Asplenium officinarum 1* | O | EB, # 707 | Van De Riet | This paper | HQ676516 |
| *Asplenium officinarum 2* | O | BM | Vogel | - | AF240658 |
| *Asplenium officinarum 3* | O | BM | - | 22 | AF516256 |
| *Asplenium officinarum 4* | O | BM | - | 22 | AF516257 |
| *Asplenium onopteris 1* | O | BM, Schneider s.n. | Schneider | 4 | AY300078 |
| *Asplenium onopteris 2* | O, N | BM, ONO-101 |  | This paper | HQ676541 |
| *Asplenium ruta-muraria 1* | O, N | EB, #712 | De Groot | This paper | HQ676517 |
| *Asplenium ruta-muraria 2* | O | BM, RUT-16 | Vogel | 3 | AF525242 |
| *Asplenium saggitatum 1* | O | BM | Vogel | This paper | AF240661 |
| *Asplenium saggitatum 2* | O | BM, SAG-1 | Vogel | 3 | AF525261 |
| *Asplenium scolopendrium 1* | O | EB, #309 | De Groot | This paper | HQ676518 |
| *Asplenium scolopendrium 2* | O | BM, SCOL-73 | Vogel | 3 | AF525262 |
| *Asplenium scolopendrium 3* | O | BM | Vogel | - | AF240660 |
| *Asplenium septentrionale 1* | O | BM, SEPT-17 | Vogel | 3 | AF525248 |
| *Asplenium trichomanes ssp. quadrivalens 1* | O | BM, Gilman 01185 | Gilman | 7 | AY549870 |
| *Asplenium trichomanes ssp. quadrivalens 2* | O | BM, Q-272 | Vogel | 7 | AY549847 |
| *Asplenium trichomanes ssp. trichomanes* | O | ISC, Shaw 20 | Shaw | 7 | AY549864 |
| *Asplenium trichomanes ssp. inexpectans* | O | BM, I-88H | Vogel | 7 | AY549868 |
| *Asplenium viride 1* | O | BM, 293A | - | 23 | EF645599 |
| *Asplenium viride 2* | O | BM | Vogel | - | AF240664 |
| *Asplenium viride 3* | O | BM, 272C | - | 23 | EF645603 |
| *Athyrium distentifolium* | O | BM, DIST-3 | Vogel | 4 | AY300047 |
| *Athyrium filix femina 1* | O, N | EB, #306 | De Groot | This paper | HQ676519 |
| *Athyrium filix femina 2* | G | - | - | 9 | EU329076 |
| *Blechnum spicant 1* | O, N | EB, #911 | De Groot | This paper | HQ676520 |
| *Blechnum spicant 2* | G | H, #1127661 | Korpelainen | 24 | EF427640 |
| *Botrychium lunaria* | G | NCU, Hauk 564 | Hauk | 25 | AY138430 |
| *Botrychium matricariifolium* | G | - | - | - | DQ849155 |
| *Cyrtomium falcatum 1* | O, N | EB, #972 | De Groot | This paper | HQ676521 |
| *Cyrtomium falcatum 2* | G | VT, D.P.Little 342 | Little | 26 | EF177268 |
| *Cyrtomium falcatum 3* | G | KUN, LJM 059 | Lu | 13 | AY736332 |
| *Cyrtomium fortunei* | G | KUN, LJM 027 | Lu | 13 | AY736348 |
| *Cystopteris fragilis* | O, N | EB, #912 | De Groot | This paper | HQ676522 |
| *Dryopteris aemula* | G | - | - | 14 | AY268816 |
| *Dryopteris affinis 1* | O, N | EB, #706 | De Groot | This paper | HQ676523 |
| *Dryopteris affinis 2* | G | COLO, 11824 | Crabbe | 14 | AY268780 |
| *Dryopteris borreri* | G | COLO | Vasak | 14 | AY268778 |
| *Dryopteris carthusiana 1* | O, N | EB, #1317 | De Groot | This paper | HQ676524 |
| *Dryopteris carthusiana 2* | G | - | - | 14 | AY268818 |
| *Dryopteris carthusiana 3* | G | COLO, 9327 | Argus | 14 | AY268777 |
| *Dryopteris cristata* | O, N | TUR, Christenhusz 3764 | Christenhusz | This paper | HQ676525 |
| *Dryopteris dilatata 1* | O, N | EB, #1315 | De Groot | This paper | HQ676526 |
| *Dryopteris dilatata 2* | G | COLO, 679 | Krasnobovov | 14 | AY268779 |
| *Dryopteris expansa* | G | COLO, 7921 | Nelson | 14 | AY268775 |
| *Dryopteris filix mas 1* | O, N | EB, #305 | De Groot | This paper | HQ676527 |
| *Dryopteris filix mas 2* | G | COLO, 1421 | Hogan | 14 | AY268776 |
| *Dryopteris oreades* | G | COLO | Vasak | 14 | AY268781 |
| *Dryopteris submontana* | O | BM | Vogel | - | AF240671 |
| *Gymnocarpium dryopteris 1* | O, N | EB, #304 | De Groot | This paper | HQ676528 |
| *Gymnocarpium robertianum* | O, N | EB, #472 | De Groot | This paper | HQ676529 |
| *Ophioglossum vulgatum 2* | G | K, Burrows 5752 | Burrows | 25 | AY138450 |
| *Oreopteris limbosperma* | O, N | TUR, Christenhusz 3719 | Christenhusz | This paper | HQ676530 |
| *Osmunda regalis 1* | G | - | - | 27 | AY651837 |
| *Osmunda regalis 2* | G | GOET, Schwertfeger s.n. | Schwertfeger | 28 | EF588817 |
| *Osmunda regalis 3* | G | TUR, Christenhusz 4271 | Christenhusz | 28 | EF588815 |
| *Phegopteris connectilis 1* | O, N | EB, #308 | De Groot | This paper | HQ676531 |
| *Phegopteris connectilis 2* | G | UC, Cranfill s.n. | Cranfill | 19 | AF425139 |
| *Polypodium cambricum* | O | GOET, Schwertfeger s.n. | Schwertfeger | 20 | FJ825689 |
| *Polypodium interjectum* | O, N | BM, Christenhusz 390 | Christenhusz | This paper | HQ676532 |
| *Polypodium vulgare 1* | O, N | EB, #711 | De Groot | This paper | HQ676533 |
| *Polypodium vulgare 2* | O | GOET, Schneider s.n. | Schneider | 21 | EF551119 |
| *Polystichum aculeatum* | O, N | EB, #303 | De Groot | This paper | HQ676534 |
| *Polystichum setiferum 1* | O, N | EB, #700 | De Groot | This paper | HQ676535 |
| *Polystichum setiferum 2* | G | VT, ex. hort. | - | 26 | EF177316 |
| *Polystichum lonchitis 1* | O, N | EB, #919 | De Groot | This paper | HQ676536 |
| *Polystichum lonchitis 2* | G | KUN, Zika 18981 | - | 13 | AY736354 |
| *Pteridium aquilinum 1* | O, N | EB, #908 | Larssen | This paper | HQ676537 |
| *Thelypteris palustris 1* | O, N | EB, #909 | Villaret | This paper | HQ676538 |

**References**

1. Zhang G, Zhang X, Chen Z, Liu H, Yang W (2007) First insights in the phylogeny of Asian cheilanthoid ferns based on sequences of two chloroplast markers. Taxon 56: 369-378.

2. Wolf PG, Soltis PS, Soltis DE (1994) Phylogenetic relationships of Dennstaedtioid ferns: evidence from *rbcL* sequences. Mol Phylogenet Evol 3: 383-392.

3. Pinter I, Bakker F, Barrett J, Cox C, Gibby M et al. (2002) Phylogenetic and biosystematic relationships in four highly disjunct polyploid complexes in the subgenera *Ceterach* and *Phyllitis* in *Asplenium* (Aspleniaceae). Org Divers Evol 2: 299-311.

4. Schneider H, Russell SJ, Cox CJ, Bakker F, Henderson S et al. (2004) Chloroplast phylogeny of asplenioid ferns based on *rbcL* and *trnL-F* spacer sequences (Polypodiidae, Aspleniaceae) and its implications for biogeography. Syst Bot 29: 260-274.

5. Bellefroid E, Rambe SK, Leroux O, Viane RLL (2010) The base number of ‘loxoscaphoid’ *Asplenium* species and its implication for cytoevolution in Aspleniaceae. Ann Bot 106: 157-171.

6. Schulze G, Treutlein J, Wink M (2001) Phylogenetic relationships between *Asplenium bourgaei* (Boiss.) Milde and *A. jahandiezii* (Litard.) Rouy inferred from morphological characters and *rbcL* sequences. Plant Biol 3: 364-371.

7. Schneider H, Ranker TA, Russell SJ, Cranfill R, Geiger JM et al. (2005) Origin of the endemic fern genus *Diellia coincides* with the renewal of Hawaiian terrestrial life in the Miocene. Proc Biol Sci 272: 455-460.

8. Schuettpelz E, Pryer KM (2007) Fern phylogeny inferred from 400 leptosporangiate species and three plastid genes. Taxon 56: 1037-1050.

9. Adjie B, Takamiya M, Ohta M, Ohsawa TA, Watano Y (2008) Molecular phylogeny of the Lady fern genus Athyrium in Japan based on chloroplast *rbcL* and *trnL-trnF* sequences. Acta Phytotax Geobot 59: 79-95.

10. Hauk WD (1995) A molecular assessment of species relationships among cryptic species of *Botrichium* subgenus *Botrychium* (Ophioglossaceae). Am Fern J 85: 375-394.

11. Schuettpelz E, Schneider H, Huiet L, Windham MD, Pryer KM (2007) A molecular phylogeny of the fern family Pteridaceae: assessing overall relationships and the affinities of previously unsampled genera. Mol Phylogenet Evol 44: 1172-1185.

12. Little DP, Barrington DS (2003) Major Evolutionary Events in the Origin and Diversification of the fern genus *Polystichum* (Dryopteridaceae). Am J Bot 90: 508-514.

13. Lu JM, Li DZ, Gao LM, Cheng X, Wu D (2005) Paraphyly of *Cyrtomium* (Dryopteridaceae): evidence from *rbcL* and *trnL-F* sequence data. J Plant Res 118: 129-135.

14. Geiger JMO, Ranker TA (2005) Molecular phylogenetics and historical biogeography of Hawaiian *Dryopteris* (Dryopteridaceae). Mol Phylogenet Evol 34: 392-407.

## 15. Hennequin S, Schuettpelz E, Pryer KM, Ebihara A, Dubuisson J-Y (2008) Divergence Times and the Evolution of Epiphytism in Filmy Ferns (Hymenophyllaceae) Revisited. Int J Plant Sci 169: 1278-1287.

16. Tsutsumi C, Kato M (2006) Evolution of epiphytes in Davalliaceae and related ferns. Bot J Linn Soc 151: 495-510.

17. Gastony GJ, Ungerer MC (1997) Molecular systematic and a revised taxonomy of the onocleoid ferns (Dryopteridaceae: Onocleeae). Am J Bot 84: 840-849.

18. He-Nigren X, Juslen A, Ahonen I, Glenny D, Piippo S (2006) Illuminating the evolutionary history of liverworts (Marchantiophyta)--towards a natural classification. Cladistics 22: 1-31.

19. Smith AR, Cranfill RB (2002) Intrafamilial relationships of the thelypteroid ferns (Thelypteridaceae). Am Fern J 92: 131-149.

20. Otto EM, Janssen T, Kreier HP, Schneider H (2009) New insights into the phylogeny of Pleopeltis and related Neotropical genera (Polypodiaceae, Polypodiopsida). Mol Phylogenet Evol 53: 190-201.

21. Kreier H-P, Rex M, Weising K, Kessler M, Smith AR et al. (2008) Inferring the diversification of the epiphytic fern genus *Serpocaulon* (Polypodiaceae) in South America using chloroplast sequences and amplified fragment length polymorphisms. Plant Syst Evol 274: 1-16.

22. Trewick SA, Morgan-Richards M, Russell SJ, Henderson S, Rumsey FJ et al. (2002) Polyploidy, phylogeography and Pleistocene refugia of the rockfern *Asplenium ceterach*: evidence from chloroplast DNA. Mol Ecol 11: 2003-2012.

23. James KE, Schneider H, Ansell SW, Evers M, Robba L et al. (2008) Diversity arrays technology (DArT) for pan-genomic evolutionary studies of non-model organims. PLoS One 3: e1682.

24. Korpelainen H, Pietiläinen M (2008) Effort to reconstruct past population history in the fern *Blechnum spicant*. J Plant Res 121: 293-298.

25. Hauk WD, Parks CR, Chase MW (2003) Phylogenetic studies of Ophioglossaceae: evidence from *rbcL* and *trnL-F* plastid DNA sequences and morphology. Mol Phylogenet Evol 28: 131-151.

26. Driscoll HE, Barrington DS (2007) Origin of Hawaiian Polystichum (Dryopteridaceae) in the context of a world phylogeny. Am J Bot 94: 1413-1424.

27. Quandt D, Mueller K, Stech M, Hilu KW, Frey W et al. (2004) Molecular evolution of the chloroplast trnL-F region in land plants. In: Goffinet B, Hollowell V, Magill R. eds. *Monographs in systematic botany: molecular systematic of bryophytes.* St. Louis: Missouri Botanical Garden Press. pp. 13-37.

28. Metzgar JS, Skog JE, Zimmer EA, Pryer KM (2008) The paraphyly of Osmunda is confirmed by phylogenetic analyses of seven plastid loci. Syst Bot 33: 31-36.
