# Supplementary material for: Use of rbcL and trnL-F as a Two-Locus DNA Barcode for Identification of NW-European Ferns: An Ecological Perspective
Source: PLoS One. 2011 Jan 26;6(1):e16371. doi: 10.1371/journal.pone.0016371 (PMC3027654; doi:10.1371/journal.pone.0016371)
Supplement: Table S2 — Maximal intraspecific genetic distances, minimal genetic distances towards the nearest neighbour and their ratio. Data are presented for all taxa with multiple individuals in the dataset. Ratios are calculated as maximal interspec. distance/minimal intraspec. distance. All distances are uncorrected P-distances based on the combined rbcL and trnL-F sequence data. N = number of individuals in the dataset. (DOC) [file pone.0016371.s002.doc]

|  | **Intraspecific distance** | | **Interspecific distance** | |  |
| --- | --- | --- | --- | --- | --- |
| **Species** | Max. distance | N | Min. distance | Nearest neighbour | **Ratio** |
| *A. onopteris* s.l. | 0,0098 | 4 | 0,0260 | *A. septentrionale* | 2,7 |
| *A. fontanum* s.l. | 0,0019 | 3 | 0,0252 | *A. septentrionale* | 13,2 |
| *A. officinarum* | 0,0096 | 4 | 0,0780 | *A. saggitatum* | 8,1 |
| *A. ruta muraria* | 0,0061 | 2 | 0,0930 | *A. onopteris* s.l. | 15,2 |
| *A. saggitatum* | 0,0030 | 2 | 0,0100 | *A. scolopendrium* | 3,3 |
| *A. scolopendrium* | 0,0060 | 3 | 0,0100 | *A. saggitatum* | 1,7 |
| *A. septentrionale* | 0,0010 | 2 | 0,0200 | *A. ruta-muraria* | 20,0 |
| *A. trichomanes ssp. inexpectans* s.l. | 0,0042 | 3 | 0,0055 | *A. trichomanes ssp. trichomanes* | 1,3 |
| *A. viride* | 0,0032 | 3 | 0,0240 | *A. trichomanes ssp. trichomanes* | 7,5 |
| *A. filix femina* | 0,0031 | 2 | 0,0120 | *A. distentifolium* | 3,9 |
| *B. spicant* | 0,0000 | 2 | 0,0500 | *M. struthiopteris* | ∞ |
| *D. oreades* s.l. | 0,0023 | 5 | 0,0105 | *D. borreri* | 4,6 |
| *D. carthusiana* s.l. | 0,0013 | 4 | 0,0077 | *D. dilatata* | 5,9 |
| *D. dilatata* | 0,0000 | 2 | 0,0040 | *D. expansa* | ∞ |
| *G. dryopteris* | 0,0031 | 2 | 0,0140 | *D. robertianum* | 4,5 |
| *M. struthiopteris* | 0,0016 | 2 | 0,0340 | *O. sensibilis* | 21,3 |
| *O. vulgatum* | 0,0000 | 2 | 0,0970 | *B. lunaria* | ∞ |
| *O. regalis* | 0,0016 | 3 | 0,1300 | *H. tunbrigense* | 81,3 |
| *P. connectilis* | 0,0036 | 2 | 0,0410 | *O. sensibilis* | 11,4 |
| *P. globulifera* | 0,0000 | 2 | 0,1300 | *P. aquilinium* | ∞ |
| *P. vulgare* | 0,0000 | 2 | 0,0180 | *P. cambricum* | ∞ |
| *P. lonchitis* | 0,0000 | 2 | 0,0153 | *P. setiferum* s.l. | ∞ |
| *P. setiferum* s.l. | 0,0011 | 3 | 0,0153 | *P. lonchitis* | 13,9 |
| *P. aquilinum* | 0,0048 | 3 | 0,0770 | *P. interjectum* | 16,0 |
| *T. palustris* | 0,0075 | 2 | 0,0403 | *O. limbosperma* | 5,4 |
