# Supplementary material for: Use of rbcL and trnL-F as a Two-Locus DNA Barcode for Identification of NW-European Ferns: An Ecological Perspective
Source: PLoS One. 2011 Jan 26;6(1):e16371. doi: 10.1371/journal.pone.0016371 (PMC3027654; doi:10.1371/journal.pone.0016371)
Supplement: Table S3 — Used species complexes. Overview of species merged into a single complex because of identical chloroplast genomes. All sequences were named after the diploid cpDNA donor, unless this donor could not be included (Dryopteris “semicristata”, see text). (DOC) [file pone.0016371.s003.doc]

| **Complex name:** | **Included (sub-)species:** | **Ploidy:** |
| --- | --- | --- |
| *Dryopteris oreades* s.l. | *D. oreades D. affinis D. filix-mas* | *2n 4n 4n* |
| *Dryopteris carthusiana* s.l. | *D. carthusiana D. cristata* | *4n 4n* |
| *Polystichum setiferum* s.l. | *P. setiferum P. aculeatum* | *2n 4n* |
| *Asplenium trichomanes* ssp. *inexpectans* s.l. | *A. trichomanes* ssp. *inexpectans*  *A. trichomanes* ssp. *quadrivalens* | *2n 4n* |
| *Asplenium onopteris* s.l. | *A. onopteris A. adiantum-nigrum* | *2n 4n* |
| *Asplenium fontanum* s.l. | *A. fontanum*  *A. foreziense* | *2n 4n* |
